# Management and Treatment of Obsessive-Compulsive Disorder (OCD): A Literature Review

**DOI:** 10.7759/cureus.60496

**Published:** 2024-05-17

**Authors:** Kawther N Elsouri, Samantha E Heiser, Dominick Cabrera, Sami Alqurneh, Jaime Hawat, Michelle L Demory

**Affiliations:** 1 Osteopathic Medicine, Nova Southeastern University Dr. Kiran C. Patel College of Osteopathic Medicine, Fort Lauderdale, USA; 2 Osteopathic Medicine, William Carey University College of Osteopathic Medicine, Hattiesburg, USA; 3 Medicine, Nova Southeastern University Dr. Kiran C. Patel College of Allopathic Medicine, Fort Lauderdale, USA; 4 Allopathic Medicine, Nova Southeastern University Dr. Kiran C. Patel College of Allopathic Medicine, Fort Lauderdale, USA; 5 Immunology, Nova Southeastern University Dr. Kiran C. Patel College of Allopathic Medicine, Fort Lauderdale, USA

**Keywords:** psychotherapy, selective serotonin reuptake inhibitor, ablative therapy, deep brain stimulation, anxiety disorders, psychosurgery, obsessions, compulsion, cognitive behavioural therapy (cbt), obsessive-compulsive disorder (ocd)

## Abstract

Obsessive-compulsive disorder (OCD) is a prevalent and debilitating mental health condition. This literature review examines the latest strategies in managing and treating OCD, with an emphasis on psychotherapy, pharmacological interventions, and neurosurgical options. A comprehensive literature search utilizing PubMed, Google Scholar, ClinicalKey, and Embase databases was conducted. Utilizing chosen keywords, the resulting articles were filtered based on inclusion and exclusion criteria. Included articles were used to discuss current research regarding OCD treatment and management. Findings reveal the efficacy and obstacles of treatments such as cognitive-behavioral therapy, selective serotonin reuptake inhibitors (SSRIs), serotonin-norepinephrine reuptake inhibitors (SNRIs), and evidence-based neurosurgical methods, offering a broad perspective on OCD management. We discuss the limitations of these established treatments and examine the innovative response of neurosurgery in treating patients with OCD. This review highlights the importance of individualized treatment plans and areas for future research.

## Introduction and background

**Figure 1:**
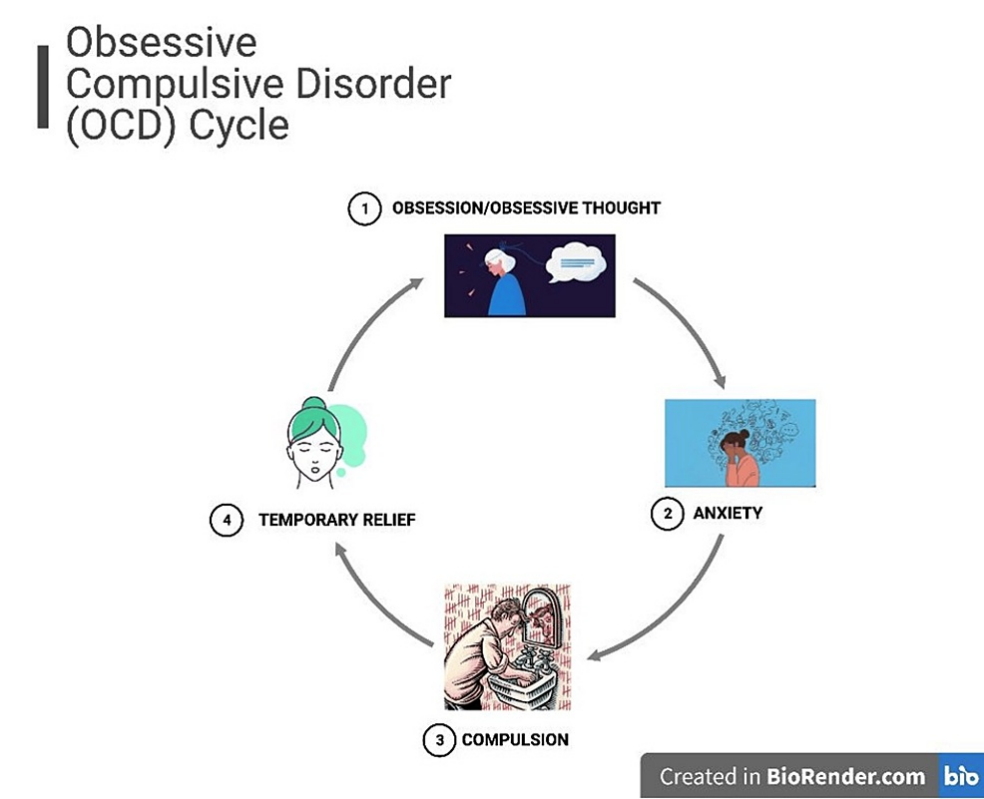
Visual representation of the repetitive cycle of OCD An obsession/obsessive thought (1) builds up to overwhelming anxiety (2), leading to an attempt to satisfy the obsessive thought (otherwise known as a compulsion) (3) and briefly providing relief (4) before the onset of another obsession/obsessive thought (1). OCD: Obsessive-compulsive disorder **Original image created with BioRender.com**

The American Psychiatric Association (APA) defines obsessive-compulsive disorder (OCD) as a psychiatric illness consisting of obsessions and compulsions [1]. Obsessions are persistent thoughts, urges, or images that can cause severe anxiety or distress [1]. Compulsions (or rituals) are repetitive behaviors, or mental acts, performed in response to the obsessions and to reduce the distress or anticipated consequence [1]. Obsessions and compulsions can range from intrusive thoughts of contamination, leading to prolonged periods of handwashing, to a fear of abandonment, resulting in hoarding behaviors [1]. In general, obsessions and compulsions are time-consuming, distressing, and often resistant to efforts to suppress them [1] (Figure 1).

The Diagnostic and Statistical Manual of Mental Disorders, Fifth Edition (DSM-5) [2], and other standard diagnostic classifications, such as the International Classification of Diseases, Tenth Edition (ICD-10) [3], categorize and identify OCD as an isolated classification. However, symptoms used to define OCD are diverse and include a range of intrusive thoughts, preoccupations, rituals, and compulsions [4]. Two individuals with OCD may exhibit entirely different manifestations and presentations of the disorder [4].

**Figure 2:**
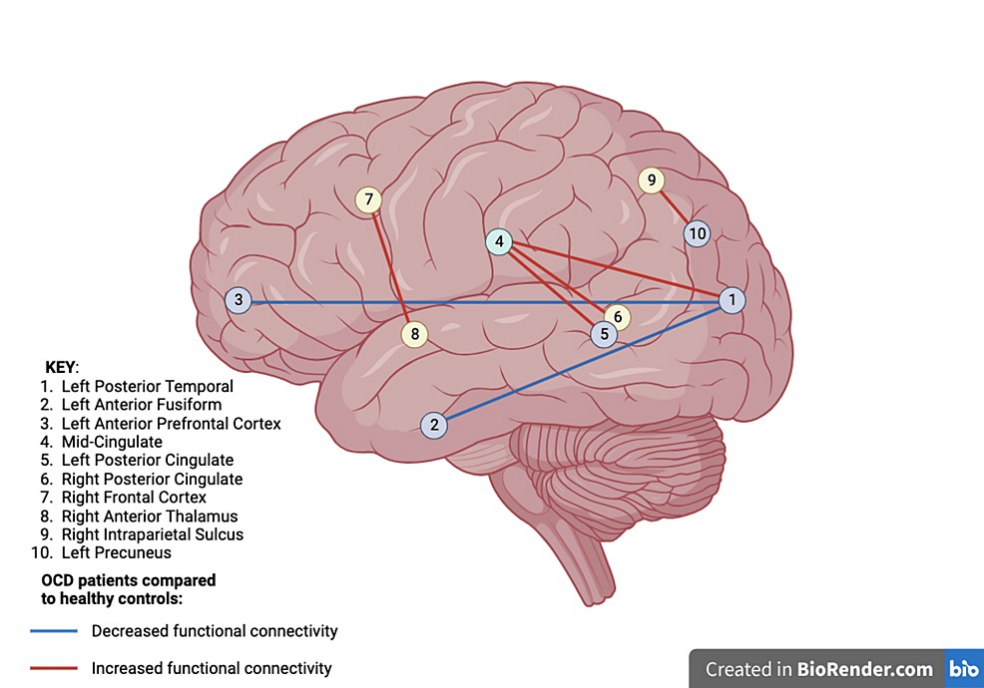
Visual representation of functional neural connectivity in active OCD Alterations in neural connectivity are reportedly involved in active OCD compared to healthy controls [6,7]. OCD: Obsessive-compulsive disorder **Original image created with BioRender.com**

Over time, distinct subtypes have been colloquially utilized to categorize OCD. These include obsessions focused on, but not limited to: (1) contamination, (2) symmetry/ordering, (3) taboo thoughts, and (4) responsibility/fear [5]. It is now believed that each subtype may be associated with a unique set of underlying psychological and biological mechanisms [5] (Figure 2).

The average age of symptom onset ranges between 22 and 36 years old [8]. Affected patients spend an average of 17 years before receiving a correct diagnosis and treatment, and with delayed diagnosis, there is an increase in disease severity as symptoms are left untreated [8,9]. Timely and effective treatment is vital in the management of OCD, particularly given the detrimental outcomes associated with uncontrolled disease. Individuals with OCD face a notably increased risk of death due to both natural and unnatural causes compared to the general population [10]. Among the unnatural causes of death, such as suicide, a recent systematic review and meta-analysis reported that individuals with OCD have a mean lifetime suicide attempt of 14.2%. This mean is significantly higher than those observed in the general population [11]. Cognitive behavioral therapy (CBT), especially exposure and response prevention (ERP), is known as a primary treatment, demonstrating substantial effectiveness in alleviating OCD symptoms. Pharmacologic interventions, such as selective serotonin reuptake inhibitors (SSRIs) and serotonin-norepinephrine reuptake inhibitors (SNRIs), are well-established in their efficacy and are frequently used either as standalone treatments or in conjunction with psychotherapy. Nonetheless, studies report that up to 60% of patients with OCD do not experience relief from these methods of treatment [12]. The variability in response to these interventions has led to other proposed methods for treatment management. Surgical modalities represent a frontier in OCD treatment, with ongoing studies focusing on patient selection and optimizing stimulation sites to address specific aspects of this multifaceted disorder [13]. This review aims to examine psychotherapeutic, pharmacologic, and neurosurgical treatments for OCD, including their advantages and limitations considering recent literature, highlighting the critical role of personalized approaches in effectively treating this complex disorder.

## Review

Methods

A comprehensive search was conducted utilizing PubMed, Google Scholar, ClinicalKey, and Embase search engines. Keywords applied included: "obsessive compulsive disorder", "OCD", "treatment", "therapy", "psychotherapy", "pharmacology”, "psychopharmacology”, "obsessive compulsive disorder and SSRI", "refractory obsessive compulsive disorder”, "refractory OCD", "OCD surgical treatment", "transcranial magnetic stimulation in OCD", "deep brain stimulation in OCD", and "electroconvulsive therapy in OCD". The inclusion criteria involved: patients diagnosed with OCD based on the DSM-5 and the ICD-10 criteria, human subjects, various OCD treatments ranging from psychotherapy to SSRIs and SNRIs as monotherapy or combination therapy, patients who underwent neurosurgical procedures specifically for OCD, literature reviews, case reports, randomized control trials, and articles published in English. The exclusion criteria were set to omit studies involving animal subjects, articles not published in English, patients with obsessive-compulsive-related disorders (OCRD), cases of OCD induced by viral or bacterial infections, and articles published prior to 2004. Our selection process was aimed at collating the most recent and pertinent studies, providing a comprehensive overview of the current treatment paradigms in OCD.

Psychotherapy

Due to the feelings of shame and guilt associated with experiencing OCD symptoms, many individuals do not receive pharmacologic treatment until years after the onset of symptoms. Existing evidence suggests that less than 10% of patients under a clinician’s care receive evidence-based treatment [11]. Lack of knowledge about the disorder, embarrassment from symptoms, and anxiety related to triggers can all contribute to the delay in patients seeking treatment [11]. Motivational interviewing techniques focus on empathizing with patients’ experiences and helping them identify and change maladaptive behaviors by meeting them at their stage of readiness to change. Patients are encouraged to recognize the disparities between their actual behaviors and their broader value system while enhancing their sense of self-efficacy to correct certain behaviors. Effective motivational interviewing typically involves one to five sessions, each lasting approximately an hour [14]. A patient’s treatment preferences should be explored to identify the most feasible, affordable, and efficacious modality for their unique circumstance.

CBT aims to rectify distorted and maladaptive beliefs to achieve symptom reduction and improved functioning using techniques such as education, relaxation techniques, coping skills training, stress management, and assertiveness training [10]. ERP is a cognitive reappraisal and behavioral intervention that has been demonstrated as the preferred treatment for OCD [15]. During ERP in CBT (CBT+ERP), individuals are guided to refrain from compulsive behaviors while gradually facing fear-provoking stimuli for prolonged periods of time [16]. A recent meta-analysis highlighted CBT+ERP’s consistent effectiveness in treating OCD among adults and children. This integration of ERP with cognitive elements exhibited a capacity to reduce symptom severity and patients' resistance to treatment [15]. Particularly noteworthy is ERP's effectiveness for patients with poor insight and low tolerance for exposure, often accompanied by discussion of feared consequences and dysfunctional beliefs [16]. 

However, it is important to consider that CBT, on its own, can still have a significant impact on OCD symptom severity. Using Yale-Brown Obsessive-Compulsive Scale (YBOCS) scores, one study demonstrated a noteworthy improvement in self-reported symptom severity among OCD patients who adhered to four weeks of intensive (and strictly adherent) CBT [17]. The YBOCS involves patients self-reporting a standardized symptom checklist and assigning scores to the severity of obsession and compulsion features. This scale is widely used to measure treatment response. A recent publication by Hirschtritt, Bloch, and Mathews suggests the superiority of CBT over pharmacologic therapy for OCD patients [18]. Their study aimed to analyze the number needed to treat (NNT), a measure of clinical benefit that assesses the number of patients needing treatment to benefit one person. The results demonstrated that three patients would need treatment for one individual to benefit from CBT, whereas five would need treatment for one person to benefit from SSRIs. Given the potential risk of adverse events arising from untreated OCD, including suicide, these findings underscore an additional advantage of CBT compared to pharmacologic treatment. They also emphasize the urgency of timely diagnosis and the initiation of treatment to halt the progression of the disorder.

CBT offers a myriad of benefits for the patient. In a study utilizing functional magnetic resonance imaging (fMRI), a comparison of connectivity changes before and after CBT, conducted between OCD patients (N=43) and healthy controls (N=24), revealed significant neuro-connective differences [17]. fMRI is capable of detecting fluctuations in blood flow and oxygenation in activated brain regions, thereby establishing links between specific areas and their functions. Patients undergoing CBT were found to have substantial increases in neural network activity [17]. These enhancements include novel connections, such as those between the cerebellum and caudate and putamen as well as between the cerebellum and dorsolateral and ventrolateral prefrontal cortices. The advantages of these newfound connections include increased resistance to compulsions and likely signify the adoption of new goal-directed behaviors and thought patterns in individuals with OCD. 

While evidence supports CBT as a promising treatment for patients with OCD, many challenges may arise with this option. Access to healthcare providers trained in CBT remains an issue for many patients, as does adherence to prolonged treatment [15]. Patients who can access these services may also face high out-of-pocket costs and intense time requirements (typically at least an hour of weekly therapy sessions in addition to daily “homework” for at least three months).

To this day, little is known about long-term outcomes following the termination of CBT for patients with OCD [16]. Pharmacotherapy has been demonstrated to be more cost-effective than CBT, and many patients who underwent CBT in clinical trials were already being treated with pharmacotherapy [19]. The latter provides relief to certain barriers of CBT namely affordability, adherence, and incomplete treatment response.

Pharmacotherapy

The approach to treating OCD with pharmacotherapy has evolved over time to identify the most effective drugs with acceptable safety profiles. Clomipramine was the first agent shown to be effective in treating OCD [15]. Clomipramine is a tricyclic antidepressant (TCA) that works by decreasing the reuptake of many neurotransmitters (serotonin, norepinephrine, acetylcholine, etc.). While the exact mechanism of why this TCA uniquely produces an anti-obsessive effect in OCD patients has been related to its ability to imitate the actions of SSRIs and SNRIs, the exact mechanism is still widely debated [15]. This unique TCA propagates its mechanism by acting more like SSRIs and SNRIs, with diminished anti-cholinergic and histaminergic adverse effects compared to others. On the other hand, SSRIs may be a preferable choice for long-term treatment as they have demonstrated a better safety and tolerability profile compared to clomipramine [20]. Multiple double-blind placebo-controlled studies have shown that while clomipramine is effective and generally well tolerated, it may lead to side effects including seizures, increased aminotransferase levels, and increased heart rate [20].

A Cochrane review found similar efficacy among all SSRIs, which include citalopram, fluoxetine, fluvoxamine, paroxetine, and sertraline [21]. Paroxetine may be preferred when OCD is comorbid with other conditions due to its marked anxiolytic effect, reduction in phobia-related avoidance, and general improvement of quality of life [21]. Escitalopram uniquely exhibits an added benefit due to minimal drug-to-drug interaction compared to other SSRIs [20]. Patients with a family history of OCD, aggressive, sexual, and religious obsessions, orbitofrontal cortex hypometabolism, and/or right caudate nucleus hypermetabolism are more likely to respond positively to SSRI treatment [20]. Conversely, limited response to SSRI treatment is associated with hoarding behavior, poor insight, severe concurrent depression, and higher levels of disability. 

When there’s a partial response to initial SSRI treatment, practice guidelines from the APA suggest that adding on an antipsychotic is preferred over switching to a different SSRI, which should only be considered in the absence of an initial response [22]. Based on pre-clinical and clinical evidence, Denys, Zohar, and Westenberg explain how the increased activity of the mesolimbic dopaminergic system impairs the prefrontal cortex’s ability to suppress anxiety-related activation of the amygdala [23]. The addition of anti-psychotics to SSRI therapy addresses both dopaminergic and serotonergic dysregulation, making it potentially effective for patients resistant to monotherapy [23]. The World Federation of Societies of Biological Psychiatry recommends the co-administration of haloperidol or a second-generation antipsychotic (such as quetiapine, olanzapine, or risperidone) with an SSRI for the treatment of resistant OCD [20]. The discrepancies in the literature regarding the treatment of SSRI-resistant OCD reveal the uncertainty surrounding the best approach for such cases and the necessity for innovative strategies tailored to each individual’s needs. 

The statistic that 40-60% of OCD patients do not respond to SSRI treatment can be frustrating when developing a treatment plan [12]. In the continuously emerging field of precision medicine, the future of therapies for treatment-resistant patients may lie in pharmacokinetic testing, particularly in relation to the cytochrome P450 (CYP450) isozymes linked to serotonergic drug metabolism. A systematic review by Marazziti et al. examined current literature on the pharmacokinetics of SSRI drugs and OCD patients [12]. The review, with a focus on sertraline, revealed high variability in plasma sertraline concentrations, with up to 15-fold difference between patients administered the same daily dosing regimen. These differing plasma concentrations are believed to stem from the variations in the CYP isoforms among OCD patients, leading to a wide range of pharmacokinetic parameters for the OCD drug. Drummond and Robert found that treatment-resistant OCD patients metabolize or absorb sertraline rapidly, resulting in subtherapeutic blood levels [24]. In their study, patients were given supratherapeutic doses ranging from 225 mg to 400 mg per day, yet blood levels were either within or below the therapeutic range. Despite this, there was an average improvement of 43% on the YBOCS. Combined, these studies show the importance of evaluating a patient’s metabolizer status, particularly in cases of OCD treatment resistance. There remains limited data regarding the long-term use of supratherapeutic doses in treating OCD, and pharmacokinetic treatment response should be corroborated by measuring plasma levels [12]. 

SSRIs continue to take a leading role in providing some degree of relief for patients with refractory OCD. However, each patient's situation may present unique challenges that necessitate tailored treatment plans. These challenges could involve difficulties adhering to CBT or the need to add SSRIs with antipsychotics when SSRIs alone are insufficient. The intricate interplay between SSRIs, combination therapies involving SSRIs, and personalized dosing based on metabolism emphasizes the precision required to effectively treat individuals with OCD. It also highlights the array of options that may offer therapeutic relief.

Neurosurgery

Even with appropriate OCD treatment, approximately half of OCD patients continue to experience disabling residual symptoms [25]. For those with treatment-resistant or refractory OCD, neuromodulation and neurosurgery may be a viable option [15]. In this review, the term “refractory OCD” is employed to encompass both refractory and treatment-resistant OCD patients. It is defined as a lack of response after three to six months of treatment with at least three different antidepressants, including clomipramine, and at least two add-on trials involving atypical antipsychotics [25]. 

The evolution of surgical and procedural treatments for refractory OCD has come a long way since their inception in the 14^th^-18^th^ centuries [26]. During that period, therapeutic phlebotomy, which involved drawing blood from the body, was used as a treatment. This procedure was based on the belief that it could “adjust the bodily humors”, as ancient theories suggested that certain human moods, emotions, and behaviors were influenced by an excess or lack of specific body fluids, known as “humors”: blood, yellow bile, black bile, and phlegm [26,27].

During the early to mid-20th century, the term “psychosurgery” emerged as a neurosurgical treatment option for refractory OCD [28]. This approach often involved a prefrontal or transorbital lobotomy, where an instrument was inserted into the frontal lobe and blindly swept. While these procedures were often crude and had detrimental effects, they provided some of the earliest evidence showing that specific neuroanatomical areas of the brain could influence mood and behavior [29]. 

In recent years, research into refractory OCD and its underlying neurological pathways has brought the benefits of neurosurgery to the forefront. These surgical procedures target brain regions that have shown strong correlations with obsessions, mood, motivation, and behavior, as will be discussed later on. Some common techniques used today include anterior cingulotomy, subcaudate tractotomy, limbic leucotomy and anterior capsulotomy, and magnetic resonance (MR)-guided focused ultrasound [28,29]. These procedures use magnetic resonance imaging (MRI), computed tomography (CT), or ultrasound guidance to precisely target specific brain regions associated with obsessions and compulsions [29]. Anesthesia is often used to minimize pain and side effects during these surgeries. Below, we highlight common neurosurgical techniques and the anatomic areas they target.

Deep brain stimulation

For patients with OCD, deep brain stimulation (DBS) can offer a life-changing alternative. DBS involves the implantation of an electrical device into specific brain regions, such as the accumbens nucleus, anterior limb of the internal capsule, or the thalamic nucleus. These areas are targeted due to their influence on motivational and emotional processes, which can become overactive in patients with OCD [30-32]. Electricity is then sent to this device to modulate the function and responses of these areas. Personalization of DBS treatment can be achieved by adjusting the voltage used during treatment.

In a recent meta-analysis and systematic review conducted by Cruz et al., 303 patients across 25 studies were examined [33]. The review focused on pre-DBS YBOCS scores and post-DBS YBOCS scores. Their findings demonstrated that DBS had a statistically significant impact on improving obsessive-compulsive symptoms in patients with refractory OCD. Specifically, bilateral DBS of the accumbens nucleus appeared effective in reducing obsessions and compulsions. The study noted that stimulating one area within the cortico-striatal-thalamo-cortical circuit, which includes the thalamic nucleus and accumbens nucleus associated with motivation and behavior in OCD, can have distant effects on other areas within this circuit. This may explain why targeting isolated areas within the circuit can modify the function and responses of unstimulated areas, potentially increasing the treatment of refractory OCD. 

Several factors that could potentially affect the efficacy of DBS such as age, gender, age of onset of OCD, area of DBS implantation, and years of OCD duration prior to DBS implantation, have been investigated in a second meta-analysis conducted by Alonso et al. [31]. They examined 116 patients across 31 studies, utilizing the YBOCS. The analysis concluded that DBS is an efficacious procedure, as evidenced by a global percentage reduction of 45.1% in YBOCS scores, with no significant differences related to some of the aforementioned factors. They did report a positive correlation between the age of onset of OCD and the response rate to DBS treatment.

It is worth noting that the most frequent stimulation-related adverse effect observed in DBS treatment was a hypomanic state or mood disinhibition characterized by increased activity, decreased need for sleep, and distractibility [31]. This adverse effect was generally described as mild, transient, and reversible in almost all studies where it occurred. Given its multiple target sites and the positive impact it can have on the lives of individuals with refractory OCD, DBS has the potential to emerge as a prominent treatment option for patients with refractory OCD.

Transcranial magnetic stimulation

Transcranial magnetic stimulation (TMS) involves the use of electric currents pulsed through a coil to generate a magnetic field [34]. This magnetic field can penetrate the skull and modify ion currents in the brain, influencing neuron excitability in specific areas associated with compulsions, motivation, and behavior. In a meta-analysis by Trevizol et al., the effectiveness of active TMS versus sham TMS in treating OCD was assessed [35]. The study targeted several areas, including the left and right dorsolateral prefrontal cortex, supplementary motor area, and orbitofrontal cortex. These areas were selected for TMS because of their role in a person’s ability to suppress intrusive thoughts, impulses, images, and repetitive motor responses. A total of 483 patients across 15 studies contributed data for the estimation of the main outcomes, mean values, and response and remission rates. The team utilized pre- and post-treatment YBOCS scoring to measure outcomes. They concluded that active TMS was statistically superior in reducing total YBOCS scores when compared to the control group in the treatment of OCD. Both this study and a second study conducted by Ikawa et al. demonstrated that targeting the bilateral dorsal prefrontal cortex could alter a patient’s ability to control obsessions by inducing neuron activity in these targeted areas and affecting neuroplasticity [36,37].

It is important to note that TMS treatment may have some potential adverse effects, including headaches, weakness, or fatigue shortly after sessions. Fortunately, these effects typically resolve on their own within two to three hours after treatment is completed [37]. 

Electroconvulsive therapy

Electroconvulsive therapy (ECT) is often a less-considered option for individuals with refractory OCD [38,39]. ECT involves the application of electrical currents to the brain to intentionally induce a seizure [40]. This procedure is believed to modify brain chemistry and electroactivity with the aim of achieving a rapid remission of symptoms. Today, ECT is typically administered under anesthesia to minimize potential side effects, which include confusion, memory loss, muscle aches, nausea, headaches, and cardiac events [40]. While the use of ECT for refractory OCD can be a controversial treatment approach, several studies suggest its effectiveness in these patients, particularly those with comorbid conditions such as depression or OCRD. OCRD includes conditions like body dysmorphic disorder, tic disorder, and self-injurious behaviors associated with OCD [37]. A systematic review by Fontenelle et al. included data from 279 patients across 50 articles to assess the main outcomes of ECT as a treatment for OCD [37]. The outcomes measured included the post-treatment onset of OCD symptoms, depressive symptoms, and the number of ECT sessions required, among others. This review found that in 265 cases, there was a 60.4% positive response rate to ECT characterized by a delayed onset of OCD symptoms post-treatment, a higher likelihood of not being depressed, and a reduced number of ECT sessions required. 

The positive effects observed with ECT may stem from the modulation of the brain’s chemistry and electroactivity within specific regions, including the accumbens nucleus, dorsolateral prefrontal cortex, and thalamic nucleus. These neuroanatomical sites are associated with executive functions and motivational processes. ECT has the capacity to influence these functions, potentially altering automatically generated OCD impulses leading to a reduction in OCD symptoms [37].

In a second systematic review by Dos Santos-Ribeiro et al., a closer examination was made regarding the application of ECT in the context of OCRDs [38]. The study evaluated 64 patients who had at least one type of OCRD and had undergone ECT. Multiple scales were employed, including the modified YBOCS for body dysmorphic disorder (BDD), the Yale Global Tic Severity Scale for tic disorder, and assessments for neurotic excoriation. It was concluded that 73.4% of these patients experienced a positive response to ECT, characterized by a decrease in post-treatment YBOCS scores, Yale Global Tic Severity Scale scores, and the authors’ categorical description of clinically meaningful benefits in addressing repetitive behaviors. This positive response included 44.0% of patients with BDD, 74.1% with tic disorders, and 85.7% of self-injurious behaviors. It is important to note, however, that relapse occurred in approximately 55.6% of these patients.

Both systematic reviews highlighted a significant limitation in the existing research: the absence of randomized controlled trials for the use of ECT in refractory OCD and OCRDs. They also noted that follow-up evaluations for these cases were often unattainable, as less than 40% of the OCRD patients followed up post-treatment. This loss of data may have a substantial impact on the results and interpretation, potentially altering the balance between positive responders and non-responders, which in turn could affect the assessment of ECT’s effectiveness in treating refractory OCD and ORCD [37,38]. ECT therapy would therefore highly benefit from future random controlled trials and research investigating its use in the treatment of refractory OCD.

Ablative surgery

An exciting emerging treatment option for refractory OCD is ablative surgery. The primary goal of ablative surgery in the context of OCD is to remove or disrupt specific brain regions associated with OCD, such as the anterior limb of the internal capsule and the anterior cingulate cortex. In a study conducted by Spatola et al., patients with refractory OCD underwent gamma knife radiosurgery to ablate a portion of the midputaminal point of the anterior limb of the internal capsule [41]. This targeted site was chosen due to its known involvement in decision-making, cognitive processing, motivation, and emotion regulation [41-44]. The study involved pre- and post-surgical assessments of YBOCS scores and neuropsychiatric evaluations. The results revealed a significant reduction in YBOCS scores after the gamma knife radiosurgery compared to pre-surgical scores.

A study by Hageman et al. yielded similar results for ablative surgery [30]. Based on the collected data, it was concluded that ablative surgery offers effective treatment options for individuals with refractory OCD, and they are generally well-tolerated with minimal post-surgical side effects, such as memory changes, loss of interest, weight gain, and personality changes [36]. As further research and trials are conducted in the field of ablative surgery, its potential as a treatment option is likely to gain more recognition and could become a leading approach in the management of refractory OCD.

## Conclusions

The ever-expanding landscape of medical therapies has offered diverse treatment options for OCD patients. Given the complex and varied nature of OCD presentations, it is crucial that treatment methods are equally diverse. Current literature supports the effectiveness of addressing treatment-refractory OCD through pharmacotherapy, behavioral therapy, and neurosurgery, whether as standalone treatments or in various combinations. CBT has proven effective for OCD, especially when combined with SSRIs, but it requires commitment and diligence from patients. The use of pharmacogenetic testing to personalize therapeutic doses can help with resistant cases. For patients who remain treatment-refractory, neurosurgery options such as DBS, TMS, ECT, and ablative surgery may offer a ray of hope. While these surgical interventions hold great potential for the future of psychiatric relief, further studies, including larger cohorts and longitudinal research, are necessary to establish their efficacy and safety. Overall, the evolving landscape of OCD treatments provides optimism, with the potential for more effective and personalized approaches to help individuals with this challenging disorder.
